# Belly flap from Norwegian spring-spawning herring (*Clupea harengus L.)*: A potentially new product with high content of vitamin D, EPA and DHA

**DOI:** 10.1016/j.heliyon.2020.e05239

**Published:** 2020-10-16

**Authors:** Margareth Kjerstad, Wenche Emblem Larssen, Lisa Kolden Midtbø

**Affiliations:** Møreforsking AS, 6021, Ålesund, Norway

**Keywords:** Food science, Food analysis, Nutrition, Pelagic, Rest raw material, Fatty acids, Belly flap, New product, Herring

## Abstract

Processing Norwegian spring-spawning herring (NSSH) yields large amounts of rest raw material which currently is used mainly for production of fish oil and fish meal for feed in aquaculture. However, belly flaps from NSSH might be a profitable resource as a food product. This study investigates the nutritional composition of the belly flaps.

NSSH was harvested in the main season between September and February. Chemical analyses of fatty acids, protein and vitamin A and D in belly flaps were conducted.

Belly flaps from NSSH had a mean fat and protein content of 32.3 ± 6.9 g/100 g and 12.6 ± 0.9 g/100g, respectively. Mean vitamin D level was 53.5 ± 8.5 μg/100 g and mean eicosapentaenoic acid (EPA) and docosahexaenoic acid (DHA) levels were 2.0 ± 0.8 g/100g and 2.3 ± 1.0 g/100g, respectively. Belly flap from NSSH contains twice the amount of vitamin D, EPA and DHA compared to NSSH fillet. There were large seasonal variations in the amount of both vitamin D, EPA and DHA. Mean level of vitamin A was 45.0 ± 8.9 μg/100 g. Belly flap from NSSH is a potential product for human consumption with a high nutritional value.

## 1 Introduction

**Figure 1:**
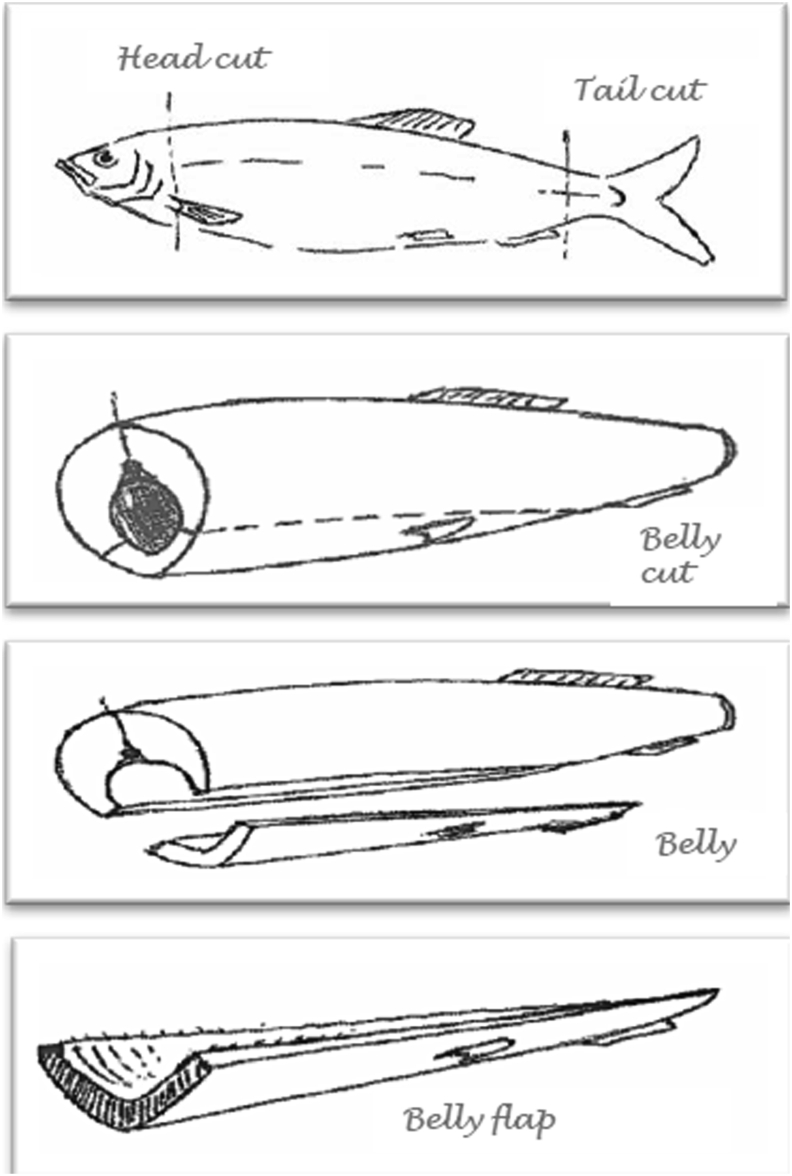
Illustration of belly flap from NSSH.

Norwegian spring-spawning herring (NSSH) is the largest herring stock in the world and has been of great importance for European fisheries (Food and Agriculture organization of the United Nations, 2018). NSSH belongs to the Atlanto-Scandian herring and is located in the North Atlantic Ocean and Barents Sea (De Barros and Holst, 1995; Holst et al., 2002). NSSH has its main spawning period during February–March (Dalpadado et al., 2000) and the main season for herring catch is from October to the beginning of March (Richardsen et al., 2019). Considering the limited catch season, a higher profit from herring fishery is of interest. In Norway, between 65-70% of the landed herring is filleted, producing a substantial amount of rest raw material. Almost 205.000 tons of rest raw material was produced from the pelagic industry in total in Norway in 2018, where most of this quantity was obtained from herring (Richardsen et al., 2019). Currently, a large degree of the rest raw material from pelagic sector is utilized, mainly for production of fish oil and fish meal as ingredients in aquaculture feed. Only a small proportion is used in high-revenue markets like nutritional supplements, cosmetics or pharmaceuticals (Richardsen et al., 2019), although there are studies evaluating the potential of rest raw material from herring, like roe and milt (Guttormsen et al., 2016). In 2014, 13% of global fish production ended up as non-food, where 76% of this was used in the production of fishmeal and fish oil used for feed in aquaculture production (European Environment Agency, 2016). Hence, the need for better utilization of rest raw material is a global issue. The Government in Norway emphasizes that improved utilization of rest raw material is essential for increased profit in the seafood industry (Minestry of Trade, Industry and Fisheries in Norway, 2019), hence there is a need for a shift in the utilization of rest raw material. The rest raw material consists of several different fractions, i.e. head, tail, gut, spine and belly flap. Belly flap is the thin, triangular-shaped area that remains after the fillet is cut from det abdomen of the fish and represent around 5–7% of the total body weight (Figure 1). Belly flap from salmon is already on the market for human consumption, with eastern-Europe and Asia as main markets (Rubin, 2011).

Herring is a rich source of n-3 fatty acids and vitamin D, and the fillet contains approximately 3 g/100g and 25 μg/100 g, respectively (Dahl et al., 2014; Seafood data, Institute of Marine Research). Health benefits connected to intake of the important long chain n-3 polyunsaturated fatty acids (LC n-3 PUFAs), are well documented (Mozaffarian and Rimm, 2006). LC n-3 PUFAs eicosapentaenoic acid (EPA, 20:5n-3) and docosahexaenoic acid (DHA, 22:6n-3) are essential for normal development of the brain and for prevention of cardiovascular disease (The European Commission, 2012; Sokola-Wysoczanska et al., 2018), and is considered to have anti-inflammatory properties (Marton et al., 2019; Simonetto et al., 2019). There is a rising demand in the marked for these fatty acids and utilisation of available sources is essential (Tocher et al., 2019). The recommended daily intake of n-3 is ≥1 E% (Nordic Council of Ministers, 2014), or 250 mg DHA and EPA for maintenance of cardiovascular health for children and healthy adults (EFSA, 2012). To reach claimed effects of maintenance of blood pressure and triglyceride levels, an intake of EPA and DHA is suggested to be between 2-4 g (EFSA, 2012).

Vitamin D deficiency is a global challenge (Michael F Holick, 2007) and vitamin D is known for being important in the maintenance of bone mineral density (Bischoff-Ferrari et al., 2004; The European Commission, 2012) and to avoid the development of rickets in children and osteoporosis in adults (M. F. Holick, 2003).Vitamin D may also be of importance for the nervous and cardiovascular system, and play a role in the immune system in addition to prevention of cancer (Hossein-nezhad & Holick, 2013; Lips, 2006; Wierzbicka et al., 2014). The daily recommended intake of vitamin D in Norway is 10 μg for persons <75 years, and 20μg > 75 years (Nordic Council of Ministers, 2014).

In addition to vitamin D, herring is considered a source for vitamin A with approximately 36 μg/100 g fillet (Seafood Data, Institute of Marine Research). Vitamin A is of importance for the maintanance of a healthy vision. Vitamin A is also a contributor for normal fetal development and for the immune system (Wiseman et al., 2017). Recommended daily intake of vitamin A in Norway is 700 μg/day for women ≥14 years old and 900 μg/day for men ≥14 years old. Pregnant and lactating women are recommended an intake of 800 and 1100 μg/100 g, respectively (Helsedirektoratet, 2014).

There is a lack of studies investigating the nutritional profile of belly flaps from NSSH, hence, the aim of this study was to investigate the nutritional content and possible seasonal variation in belly flaps from NSSH.

## 2 Material and methods

### 2.1 Belly flap sampling

NSSH were caught in the Norwegian Sea and near-shore areas of Nordland county in Norway during the commercial fishery from September 2012 to February 2013 (Table 1). Grøntvedt Pelagic (Uthaug, Norway), a Norwegian seafood company, bought herring catches from Norwegian purse seiners. To preserve the initial raw material quality, the catch was stored in refrigerated sea water giving temperatures of -1 to -2 °C until unloading to Grøntvedt Pelagic for filleting and separation of rest raw material. The rest raw material was delivered to Møreforsking AS. NSSH was processed using a BAADER 221 LA fully automated production line, including the BAADER 488 automatic feeder, BAADER 221 heading and filleting machine and BAADER 56 skinner (Baader AS, Norway). The fish varied in size, but most were between 300 to 400 g. A randomized selection of belly flaps was collected manually from the production line (n = 260). All belly flap samples were collected from the same machine. After collection, the belly flaps were cleaned manually in freshwater for removal of intestine and gonads, followed by a 20 s rinse in 5% salt brine to remove blood and other residues. Although a study has documented a catalytic effect of NaCl on lipid oxidation in fish muscle (Osinchak et al., 1992), a report from 2016 documented no accelerating effect of NaCl rinse on oxidation (Kristinova et al., 2016). The belly flaps (n = 260) were measured (weight, length and width). The average dimensions of belly flaps from NSSH were length: 17.3 ± 2.6 cm and width: 3.6 ± 0.9 cm, and the average weight was 19.9 ± 4.8 g. From the timepoints September, November and February, 100 belly flaps were collected and divided into 5 pooled parallel samples, 20 belly flaps in each sample. In January 80 belly flaps were collected and divided into 4 parallel samples (Table 1). Each pooled sample were homogenized by using a food processor (Braun Combimax 600, USA). Samples were frozen and stored at -25-30 °C. All analysis has been performed on material from the same pooled, homogenized parallel sample and approximately 1 month after the last sampling timepoint in February 2013. Prior to analysis the samples were thawed overnight at 4 °C. All sampling timepoints have n = 5 except for January with n = 4.

**Table 1:** Overview of the catches of NSSH for belly flap sampling.

Timepoint	Sampling Date	Boat name	Catch area	Sampling
September	2012.09.27	«Rogne»	FAO 27/II	100 belly flaps/5 parallells
November	2012.11.06	«Libas»	FAO 27/II	100 belly flaps/5 parallells
January	2013.01.16	«Slåtterøy»	FAO 27/II	80 belly flaps/4 parallells
February	2013.02.22	«Bluefin»	FAO 27/II	100 belly flaps/5 parallells

FAO = Food and Agriculture Organization.

### 2.2 Chemical analysis

#### 2.2.1 Water and ash content

Analysis of water and ash content were performed at Møreforsking AS by standardized methods described earlier (Synnes et al., 2007). The water content was determined by drying 5 g of the homogenized belly flaps at 105 °C until a constant weight was obtained (analytical balance: model AG204, Mettler-Toledo, Greifensee, Switzerland. Drying oven: model TS4057, Termaks, Bergen, Norway). Ash was determined by heating the homogenized samples at 550 °C until constant weight was obtained (Muffle furnace: model L9/11/06, Nabertherm GmbH, Lilienthal, Germany).

#### 2.2.2 Fat and protein content

The amount of fat and crude protein was analyzed by National Institute of nutrition and Seafood research (NIFES), Norway, now Institute of Marine Research, by accredited methods 091 and 171, respectively. The methods have both been described earlier (Tastesen et al., 2014). Total fat content was measured gravimetrically after acidic hydrolysis and petroleum ether extraction. For pre-extraction of fat, 0.7 g homogenized belly flap sample was treated with heptane. The solid residue was hydrolyzed by 4M hydrochloric acid and heptane followed by fat extraction by petroleum ether. The method has an uncertainty of 5 %. The nitrogen content was determined by the Dumas method (Leco FP-528 nitrogen analyser, Leco Corp, MI, USA). The sample (0.15–0.25 g) was burned in pure oxygen in a combustion chamber. The resulting nitrogen gas was tested for thermal conductivity in a thermal conductivity cell and total nitrogen content was measured. The amount of protein was obtained by multiplying the nitrogen content by 6.25. The method has an uncertainty of 6%.

#### 2.2.3 Fatty acid composition

Fatty acid composition was analyzed by Eurofins, Norway. The fat was extracted by chloroform and methanol following a method by Eurofins. The samples were hydrolyzed with hydrochloric acid prior to fat extraction with cyclohexane and isopropanol. Samples were vaporized followed by methylation (both acidic and alkaline) prior to separation and quantification by gas chromatography with a flame ionization detector. Further details are classified by Eurofins.

#### 2.2.4 Vitamins

Vitamin A and vitamin D were analysed by high performance liquid chromatography at Eurofins, Norway, by accredited methods. Vitamin A (all-trans-retinol and 13-cis-retinol) was analysed by method EN 12823-1 2000. Vitamin D (cholecalciferol, D_3_ and ergocalciferol, D_2_) by method BS EN 12821:2009.

### 2.3 Statistics

All data is reported as mean ± standard deviation (SD). All statistical analyses were performed by using Graphpad Software version 8.2.1 (San Diego, USA). Differences between sampling periods were tested by ANOVA followed by a multi-comparison test (Tukey HSD). Non-parametric test was performed when normality and homogeneity was not met. Correlation analysis between variables were performed by using Pearson *r* correlation. *P*-values < 0.05 were considered statistically significant. Different letters (a-d) in tables and graphs indicate statistical significance.

## 3 Results

To assess the nutritional profile of NSSH belly flaps and potential seasonal variation, samples from the different samplings (September 2012 to February 2013) were analyzed for macronutrients, fatty acids and vitamin A and D.

### 3.1 Macronutrients

**Figure 2:**
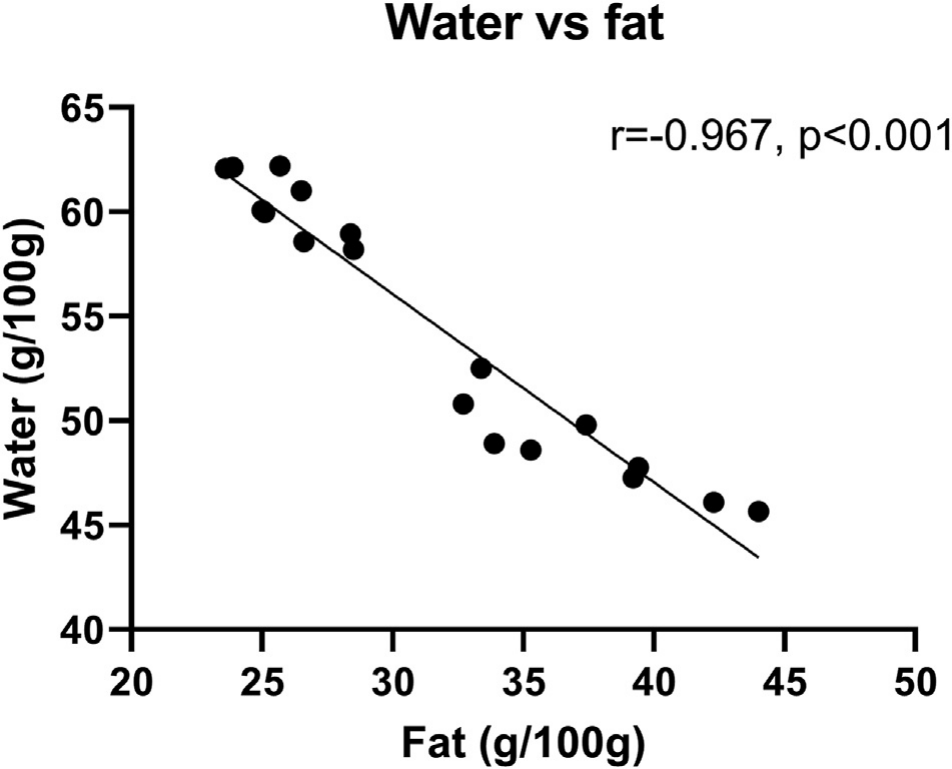
Correlation between water and total fat in NSSH belly flaps. n = 4–5.

The mean total fat content in belly flaps from NSSH from all four sampling timepoints (September–February) was 32.3 ± 6.9 g/100g with lowest amount of fat in January and February (during spawning) and highest amount in September and November (Table 2). Mean protein content was 12.6 ± 0.9 g/100 g. The highest protein level was in January and February, with 13.1 ± 0.2 and 13.5 ± 0.2 g/100g, respectively. Water content varied between the different months of sampling with the lowest amount in September (46.5 ± 0.9 g/100g) and highest amount in February (61.5 ± 1.0 g/100 g). The water content was inversely correlated with fat content (r = -0.97, p= <0.001) (Figure 2). The amount of ash varied through the year with highest amount in November with 3.3 ± 0.2 g/100g (Table 2).

**Table 2:** Macronutrient content in belly flaps from NSSH in g/100g (mean ± SD).

	n	Fat	Protein	Water	Ash
September	5	41.4 ± 2.1^a^	11.8 ± 0.8^a^	46.5 ± 0.9^a^	1.8 ± 0.1^a^
November	5	34.5 ± 1.9^b^	12.7 ± 0.8^a^	50.1 ± 1.6^b^	3.3 ± 0.2^b^
January	4	27.1 ± 1.7^c^	13.1 ± 0.2^b^	58.9 ± 0.8^c^	2.1 ± 0.0^c^
February	5	25.0 ± 1.2^c^	13.5 ± 0.2^b^	61.5 ± 1.0^d^	2.3 ± 0.1^c^
Total	19	32.3 ± 6.9	12.6 ± 0.9	54.0 ± 6.5	2.2 ± 0.6

n = 4–5. Different letters (a-d) indicate statistical significance (p < 0.05).

### 3.2 Fatty acid composition

**Figure 3:**
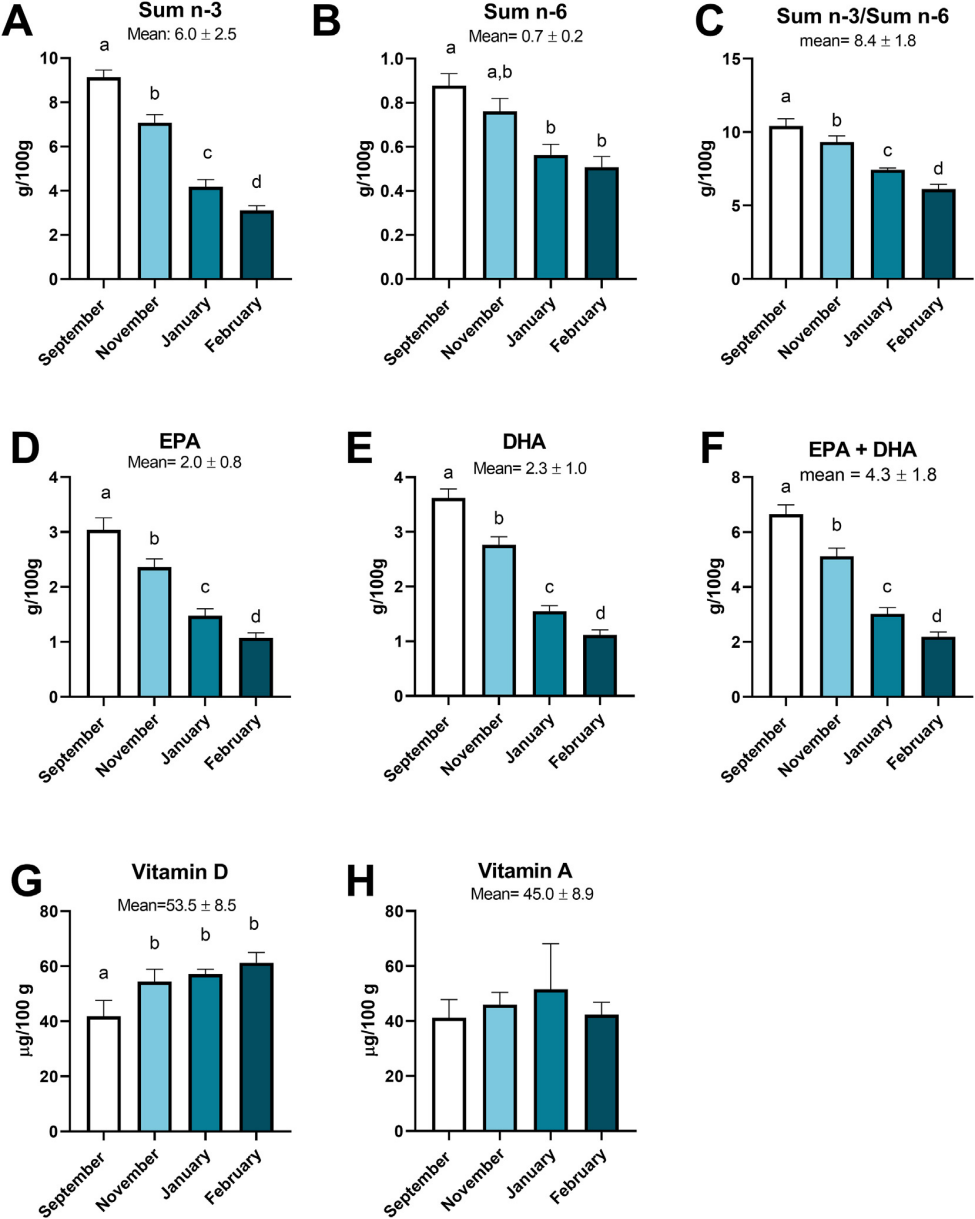
Analysis of fatty acids and vitamins in NSSH belly flaps. Results are shown as mean ± SD. Sum n-3 (A) (includes 18:3n-3, 18:4n-3, 20:3n-3, 20:4n-3, 20:5n-3, 22:5n-3, and 22:6n-3). Sum n-6 (includes 18:2n-6, 18:3n-6, 20:2n-6, 20:3n-6, 20:4n-6, 22:4n-6, 22:5n-6) (B). Sum n-3/sum n-6 (C). EPA (D). DHA (E). EPA + DHA (F). Vitamin D (G). Vitamin A (H). n = 4–5. Different letters (a–d) in tables and graphs indicate statistical significance.

The mean level of sum n-3 in belly flaps from all sampling months was 6.0 ± 2.5 g/100 g sample (Figure 3A). The level of sum n-3 was highest in September and November, with 9.1± 0.3 and 7.1 ± 0.4 g/100 g, respectively. Mean level of sum n-6 was 0.7 ± 0.2 g/100 g with the highest level in September (0.9 ± 0.1 g/100g) and November (0.8 ± 0.1 g/100g) (Figure 3B). The sum n-3/sum n-6 ratio was significantly different between sampling months with highest level in September (10.0 ± 0.5 g/100 g) (Figure 3C). Levels of EPA (Figure 3D) and DHA (Figure 3E) were significantly different between sampling months, with highest levels in September, with 3.0 ± 0.2 and 3.6 ± 0.2 g/100g, respectively. Mean level of EPA from all timepoints was 2.0 ± 0.8 g/100 g and mean DHA level was 2.3 ± 1.0 g/100g. Mean levels of EPA + DHA was 4.3 ± 1.8g/100g (Figure 3F).

### 3.3 Fat-soluble vitamins

**Figure 4:**
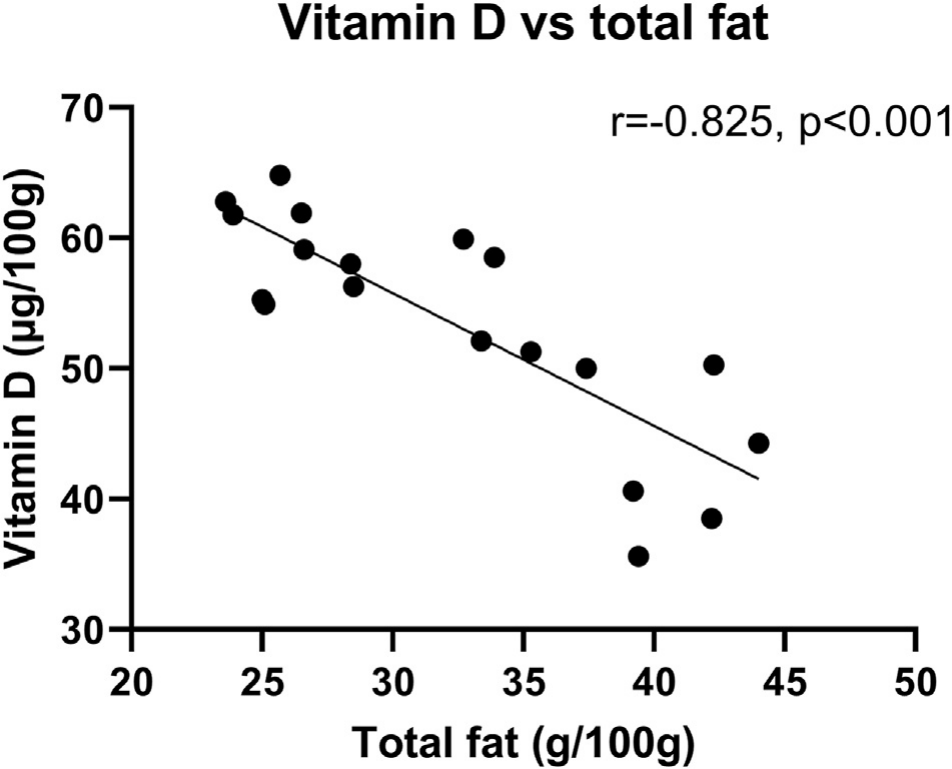
Correlation between Vitamin D and total fat in NSSH belly flaps. n = 4–5.

The content of vitamin D in the belly flaps varied between the sampling months with highest level in February (61.2 ± 3.7 μg/100g) (Figure 3G). The level of vitamin D measured in September (41.9 ± 5.7 μg/100g) was significantly lower compared with vitamin D measurements in November, January and February. There was a strong inverse correlation (r = -0.82, p < 0.001) between the amount of vitamin D vs total fat content in the belly flaps (Figure 4).

The mean level of vitamin A in belly flaps was 45.0 ± 8.9 μg/100g (Figure 3H). There was no statistical difference in the amount of vitamin A between the different timepoints. The was a large variation between the samples from the catch in January.

## 4 Discussion

Analysis of total fat in NSSH belly flap samples revealed a high fat content (32.3 ± 6.9 g/100 g), more than twice the amount compared to the amount in NSSH fillet (13.6 ± 4.6 g/100 g, n = 196) (Dahl et al., 2014). There was large seasonal variation in fat content in belly flaps, with the highest fat content during the catch period from September–November, when the herring had just arrived from the feeding area in the Norwegian Sea, and lowest in January and February, during the spawning period. This result is supported by other studies where fat content in NSSH fillet (sampled between September 2011 to January 2013) (Dahl et al., 2014) and in RRM from Atlantic herring (sampled between June 1999 and January 2001) were lowest in January to March (Aidos, van der Padt, Luten and Boom, 2002).The same fat reduction in connection with spawning is seen in both sardines (*Sardina pilchardus*), North sea herring (*Clupea harengus*) and pacific herring (*Clupea harengus pallasii*) (Bandarra et al., 1997; Frantzen et al., 2011; Huynh et al., 2007). Total fat was inversely correlated with the amount of water in the samples. This result is supported by earlier studies which have concluded that there is an inverse correlation between fat and water content in fish (Yeannes and Almandos, 2003).

Total amount of n-3 (6.0 ± 2.5 g/100 g) in NSSH belly flaps was twice the amount compared to herring fillet (approximately 3 g/100 g) (Seafood data, Institute of Marine Research). Since the mean sum of EPA + DHA was 4.3 ± 3.8 g/100 g, more than 70 % of the total amount of n-3 fatty acids in the belly flap are EPA and DHA. The amount of EPA + DHA in fillet from NSSH has earlier been measured to be only half of this amount with 2.1 g/100 g (Dahl et al., 2014). For comparison, sum EPA + DHA was 1.6 g/100g in fillet from wild Atlantic salmon, 1.2 g/100g in fillet from farmed Atlantic salmon and 4 g/100g in fillet from mackerel (Seafood data, Institiute of Marine Research).

To ensure the maintenance of general cardiovascular health, 0.25 g EPA and DHA are recommended as daily intake (EFSA, 2012). Global health organisation from EPA and DHA recommend an even higher dosage, from 0,5-1 g EPA + DHA a day to ensure a good health status (GOED, 2020). Approximately 6 g of belly flap from NSSH contains 0.25 g EPA and DHA. To achieve the daily recommended intake of 0.25 g EPA and DHA from other fish products, the intake must be approximately 13 g herring fillet, 21 g farmed and 16 g wild Atlantic salmon fillet and 7 g mackerel fillet. Hence, belly flap from NSSH is a good source for important omega-3 fatty acids.

Vitamin D level in belly flaps (53.5 ± 8.5 μg/100 g) was more than twice the amount compared to NSSH fillet, with approximately 25 ± 11 μg/100 g (n = 196) (Dahl et al., 2014). Compared to other fatty fish species, NSSH belly flap has a higher proportion of vitamin D; fillet from mackerel and Farmed Atlantic salmon contains approximately 5 μg/100 g and 7 μg/100 g vitamin D, respectively (Seafood Data, Institute of Marine Research). To achieve the daily recommended intake of vitamin D of 10 μg, the daily intake must be approximately 19 g of belly flap. In comparison, the intake of NSSH fillet must be 40 g, 200 g of mackerel and 142 g of farmed Atlantic salmon. Belly flap from NSSH is a very good source for vitamin D, which is of interest considering the global vitamin D deficiency (Holick, 2007).

Vitamin D level in belly flaps was inversely correlated with total fat content. During spawning period (January–March), the fat content was lowest, while the vitamin D content was highest. Analysis of fillets from NSSH show the same tendency with higher amount of vitamin D and lower amount of fat in the period January–March (n = 21–25) (Dahl et al., 2014). To our knowledge, there are no other literature on the relationship between the amount of fat and vitamin D in NSSH, however, the diet varies throughout the season because of the migration between spawning and feeding area (Dalpadado et al., 2000; Prokopchuk and Sentyabov, 2006), which might be a factor contributing to the seasonal variation of vitamin D in the fish. Our results suggest that NSSH belly flap is a product that has large variation in nutritional content during the year. Belly flaps had high amounts of EPA and DHA and lower amounts of vitamin D in September. In February, the vitamin D level was higher, however, EPA and DHA was lower as a result of lower fat content. This might be a factor when considering the best catch period for belly flap production. Herring is considered as its finest during September–November because of the higher fat content which gives a better texture and taste (Nielsen et al., 2005). Although vitamin D is at its lowest in September–November, it is still a rich source of vitamin D.

In NSSH belly flaps the mean vitamin A level was 45.0 ± 8.9 μg/100 g with no significant seasonal variation. This level is higher compared with results from NSSH fillet with 36 μg/100 g fillet (Seafood Data, Institute of Marine Research). To achieve the daily recommended intake of vitamin A, women and men must consume approximately 1550 g and 2000 g belly flaps per day, respectively. However, production of a high quality oil from belly flaps could yield a higher concentration of vitamin A content compared to belly flaps in its natural form. Our results indicate that belly flaps are a better source for EPA, DHA and vitamin D compared with vitamin A. Belly flap from salmon is already introduced for human consumption, with eastern-Europe and Asia as main markets (Rubin, 2011). NSSH belly flap from herring might be a potential product in markets already introduced to belly flaps from salmon. Given the high amount of vitamin D and LC n-3 PUFAs in belly flaps, this might be a motivating factor for introducing belly flaps from herring as products for human consumption. Hence, based on the nutritional value, belly flaps from NSSH have a potential of becoming a valuable product for the pelagic sector either in its natural form, or as a high-quality product or oil. Today's meal and fish oil production is based on a pooled raw material including gut, which often gives unfavorable conditions and oxidation may occur (Carvajal et al., 2015; Šližytė et al., 2014). It is possible to sort out the belly flap from the rest of the raw material before fishmeal and oil production. This will give the industry the possibility to produce a high-quality oil for human consumption, which can be used as a nutraceutical.

## 5 Conclusion

Belly flap from NSSH contains twice the amount of vitamin D and omega-3 compared with NSSH fillet, with large seasonal variations. Vitamin D level was inversely correlated with total fat content. Belly flap from NSSH has a potential of becoming a product directly for human consumption and as raw material for high-quality oil.

## Declarations

### Author contribution statement

Margareth Kjerstad: Conceived and designed the experiments; Performed the experiments; Contributed reagents, materials, analysis tools or data; Wrote the paper.

Wenche E. Larssen: Performed the experiments; Contributed reagents, materials, analysis tools or data; Wrote the paper.

Lisa K. Midtbø: Analyzed and interpreted the data; Wrote the paper.

### Funding statement

This work was supported by 10.13039/501100010197Norwegian Seafood Research Fund (FHF) (grant number: 900675).

### Competing interest statement

The authors declare no conflict of interest.

### Additional information

No additional information is available for this paper.
